# Toxic tall fescue grazing increases susceptibility of the Angus steer fecal microbiota and plasma/urine metabolome to environmental effects

**DOI:** 10.1038/s41598-020-59104-1

**Published:** 2020-02-12

**Authors:** Ryan S. Mote, Nicholas S. Hill, Joseph H. Skarlupka, ViLinh T. Tran, Douglas I. Walker, Zachary B. Turner, Zachary P. Sanders, Dean P. Jones, Garret Suen, Nikolay M. Filipov

**Affiliations:** 1 Interdisciplinary Toxicology Program, University of Georgia, Athens, GA USA; 2 Department of Physiology and Pharmacology, University of Georgia, Athens, GA USA; 3 Department of Crop and Soil Sciences, University of Georgia, Athens, GA USA; 4 Department of Bacteriology, University of Wisconsin – Madison, Madison, WI USA; 5 Department of Medicine, Division of Pulmonary, Allergy, and Critical Care Medicine, Emory University, Atlanta, GA USA

**Keywords:** Applied microbiology, Predictive markers

## Abstract

Impaired thermoregulation and lowered average daily gains (ADG) result when livestock graze toxic endophyte (*Epichloë coenophialum)*-infected tall fescue (E+) and are hallmark signs of fescue toxicosis (FT), a disease exacerbated by increased temperature and humidity (+temperature-humidity index; +THI). We previously reported FT is associated with metabolic and microbiota perturbations under thermoneutral conditions; here, we assessed the influence of E+ grazing and +THI on the microbiota:metabolome interactions. Using high-resolution metabolomics and 16S rRNA gene sequencing, plasma/urine metabolomes and the fecal microbiota of Angus steers grazing non-toxic or E+ tall fescue were evaluated in the context of +THI. E+ grazing affected the fecal microbiota profile; +THI conditions modulated the microbiota only in E+ steers. E+ also perturbed many metabolic pathways, namely amino acid and inflammation-related metabolism; +THI affected these pathways only in E+ steers. Integrative analyses revealed the E+ microbiota correlated and co-varied with the metabolomes in a THI-dependent manner. Operational taxonomic units in the families *Peptococcaceae*, *Clostridiaceae*, and *Ruminococcaceae* correlated with production parameters (e.g., ADG) and with multiple plasma/urine metabolic features, providing putative FT biomarkers and/or targets for the development of FT therapeutics. Overall, this study suggests that E+ grazing increases Angus steer susceptibility to +THI, and offers possible targets for FT interventions.

## Introduction

As global consumer interest towards pasture-based finishing production systems for beef cattle rises, a recent study estimated that, in the United States (U.S.), the current beef production capacity could only support about 27% of the current demand^1^. As such, a capacity increase of 30% would be required if there were a nationwide transition to grass-finishing production systems^1^. Considering the shift in consumer interest and the contribution of ruminant-derived methane to a warming environment, the detrimental impact of harsh environmental conditions (i.e., heat stress) on animal production efficiency, and the potential environmental impact of the necessary increase of grazing beef herds to meet future agricultural demand, there is an urgent need to optimize animal health and performance under numerous physiological stressors, including those posed by the warming environment.

Tall fescue, *Lolium arundinaceum*, covers 14 million hectares across the Southeastern U.S., and is used widely as a pasture forage in beef production systems. Wild-type tall fescue is commonly infected with the endophytic fungus *Epichloë coenophialum*, which produces secondary metabolites that improve plant vigor, thereby allowing the plant to be more tolerant of external environmental stressors (e.g., grazing, heat stress, insects)^2^. While the endophyte-infected tall fescue provides increased plant biomass^3^, the endophyte also produces toxic metabolites (e.g., ergot alkaloids). These have been linked to decreases in grazing animal health and performance, leading to a disease called fescue toxicosis (FT), which is exacerbated in harsh climates^2,4^. Thus, from an animal perspective, the wild-type endophyte-infected tall fescue is referred to as toxic (E+).

FT has multiple clinical signs, but the most economically costly, at about $1 billion per year to the U.S. beef industry, are decreased weight gain and reproductive insufficiencies^5^. Recent studies also estimate heat stress-related average annual economic losses to the U.S. animal agricultural sector at $2.4 billion, with $369 million of it accounted for by the beef industry^6^. Heat stress, however, does not act alone, and its negative effects are frequently compounded by underlying illness^7^ and/or malnutrition^8^. In this regard, important FT pathophysiological changes (e.g., impaired thermoregulatory capacity) become more profound under heat stress conditions^9–12^. This heat stress-FT interaction further complicates the development of production-restoring therapeutic and/or management strategies^13^.

Previous FT microbiota studies have focused on the effects of E+ on ruminal bacterial and fermentation profiles^14,15^. For example, one study identified ruminal hyper ammonia-producing bacteria capable of degrading the toxic ergot alkaloids^16^, indicating that these bacteria may be involved in the extensive metabolism of ergot alkaloids occurring in the rumen^17^. As ergot alkaloids, the key etiological agents of FT, are heavily metabolized in the rumen, this suggests that they might affect animal performance indirectly, i.e., through ergot alkaloid metabolites (e.g., lysergic acid) or by targeting other downstream processes responsible for the decreased animal productivity associated with FT. In this regard, we recently found that E+ grazing alters the fecal microbiota, namely affecting the *Ruminococcaceae* and *Lachnospiraceae* bacterial families under thermoneutral conditions, while also leading to a highly correlated structure of hindgut microbiota specific to E+ grazing steers^18^. Separately, under thermoneutral conditions, we reported that E+ grazing perturbs the metabolome, mainly tryptophan and tyrosine metabolism, and it does so in opposing patterns in the plasma and urine^19^. The influence of E+ grazing on the microbiota-metabolome interaction, or how elevated environmental temperature and humidity might affect the grazing beef microbiota, metabolome, and their interactions, have not been assessed thus far.

Therefore, the goals of this current study were to assess the influence of E+ grazing under thermoneutral and harsher environmental conditions on the fecal microbiota, plasma and urine metabolomes, and on the way they interact. We hypothesized that E+ grazing results in rapid and lasting perturbations of the plasma/urine metabolomes and fecal microbiota and the microbiota:metabolome interactions. As E+ steers have impaired thermoregulation, we also hypothesized that harsher environmental conditions would compound these perturbations.

## Materials and Methods

### Animal treatments and environmental conditions

All animal handling and sample collection methods were approved in advance by the Institutional Animal Care and Use Committee of the University of Georgia (A2015 11-004) and were performed in accordance with all relevant guidelines and regulations. Animals were passed through animal handling protocols and selected based on temperament and weight. Post-weaning Angus steers (n = 12; BW = 311.3 ± 5.4 kg) were blocked by weight and randomly assigned to non-toxic (Max-Q; Jesup MaxQ AR542; n = 6; 3 paddocks, 2 steers per paddock) or toxic (E+; Jesup with wild-type endophyte; n = 6; 3 paddocks, 2 steers per paddock) tall fescue treatments at the J. Phil Campbell Natural Resources Conservation Center of the University of Georgia (Watkinsville, GA) in the late Spring and early Summer of 2016 (May 11, 2016–June 6, 2016). The temperature-humidity index (THI) was used as a predictor of heat stress risk and was calculated from temperature and humidity measurements as previously described^20^. Briefly, THIs were calculated using the equation: $THI=1.8\ast \,T-(\frac{100-RH}{100})\ast \,(T-14.3)+32;$ where *T* is the ambient temperature in degrees centigrade and *RH* is the percent relative humidity. Samples were collected before and at 1, 2, 12 (low THI; −THI), 16 (high THI; +THI), 20 (+THI), and 26 (−THI) days post pasture assignment. Low THI (no heat stress risk) dates were defined as THI < 72 on two successive dates, and high THI (mild-to-moderate heat stress risk) was defined as THI > 72 for two successive dates with sample collections occurring in the afternoon on the second day. Temperature and humidity measurements were recorded from 8:00 AM–8:00 PM daily and across sampling times (8:00–11:00 AM for −THI dates, 12:00–4:00 PM for +THI dates).

### Date selection for assessment of environmental influences

To determine the effects of E+ grazing and THI on the microbiota, metabolome, and the microbiota:metabolome interactions, Day 12 (lowest −THI) and Day 20 (highest +THI) sampling dates were used as representative dates to assess potential effects of different ambient environmental conditions (i.e., −THI vs +THI) on Max-Q and/or E+ grazing steers. Further, detection of ergot alkaloids in the urine has been shown as an accurate and less variable ergot alkaloid exposure diagnostic tool^21,22^. The majority (94%) of ergot alkaloid excretion is in the urine, with ergot alkaloids appearing as early as 12 h post-exposure and concentrations being both exposure level- and duration-dependent^21^. Therefore, given that ergot alkaloids are considered the key etiological agents of FT and their urinary levels are a sensitive biomarker of ergot alkaloid exposure, in this study, ergot alkaloids in the urine reached a stable maximum and plateaued by 12 days. Urinary ergot alkaloid (UEAs) levels on Day 12 and Day 20 were significantly higher in E+ than Max-Q steers on both sampling dates (see results). Thus, Day 12 and Day 20 were used in our microbiota, high-resolution metabolomics (HRM), and the microbiota:metabolome integrative analyses to assess E+: THI interactive effects. These analyses are described later in this study.

### Urinary ergot alkaloid analysis

Total UEAs, a sensitive biomarker of exposure to E+^23^, were determined before pasture placement and at 1, 2, 12, 16, 20 and 26 days post-pasture placement via ELISA (Agrinostics Ltd. Co., Watkinsville, GA) as previously described^4,19,23^.

### Sample collection and processing

Steer body weights were recorded before pasture placement and 16, 20 and 26 days post pasture assignment with a digital scale. Fresh fecal samples were collected by hand from inside the animal using new gloves for every collection. Plasma was collected via jugular blood draw and voided urine was collected in freshly cleaned cups. Plasma, urine, and fecal matter were stored on ice for transport to the lab and long-term storage at −80 °C. Fecal, plasma, and urine samples were taken before pasture placement (Pre) and at 1, 2, 12, 16, 20, and 26 days post pasture assignment.

### DNA extraction

Fecal genomic DNA was extracted using a mechanical disruption and phenol extraction protocol established by Stevenson and Weimer^24^, with a 25:24:1 phenol:chloroform:isoamyl alcohol modification^21^. All DNA samples were resuspended in TE buffer and quantified using a Qubit® Fluorometer (Invitrogen, San Diego, CA). For each set of DNA extractions, a negative control (TE extraction buffer) was included alongside each extraction and taken through the amplification and sequencing protocols described below.

### DNA amplification and sequencing

Samples were diluted to 1 ng/µL for amplification, and universal bacterial primers for the 16S rRNA variable region V4 were used in the amplification reaction as described by Mote, *et al*.^18^. After amplification, samples were equimolar pooled into the final library, and sequenced on an Illumina MiSeq using the 2 × 250 bp paired end MiSeq v2 sequencing kit (Illumina, San Diego, CA) using custom primers^22^. The sequenced control and sample DNA were subjected to quality filtering and normalization procedures described next.

### 16S rRNA gene fecal sequence processing and bioinformatics analysis

Raw sequence files were processed using mothur v.1.38.1^25^ as in Mote, *et al*.^18^. After quality filtering, unique sequences were aligned to the SILVA version 119 reference alignment database^26^ with chimera removal (http://drive5.com/uchime). Bacterial sequences were classified using the Greengenes database v13.8 (http://greengenes.secondgenome.com)^27^. Good’s coverage was calculated in mothur, and the operational taxonomic units (OTU) were normalized for sequence depth and abundance filtered (>0.1% abundance; >50% of within-date samples) prior to statistical analysis. Community diversity was estimated using Chao1 richness^28^ and Simpson diversity index^29^; alpha diversity metrics were tested for E+ grazing and time effects utilizing the non-parametric Kruskal-Wallis test by ranks. The non-parametric permutational analysis of variance (PERMANOVA) was used to test for E+ grazing effects on the entire microbial community using both Bray-Curtis (abundance) and Jaccard (presence/absence) distance matrices, with fescue treatment and time spent grazing set as the two factors alongside the treatment by time interaction term. The same analysis was performed on day 12 (−THI) and 20 (+THI) samples using fescue treatment and THI as the two factors. Partial least squares discriminant analysis (PLS-DA) were performed, with near zero variance filtering for predictors, using the mixOmics *R* package^30,31^ on the post-sequencing depth normalized OTU table; the sequences were log transformed, mean centered and Total Sum Scale normalized prior to these analyses^30^. Linear discriminant analysis effect size (LEfSe) was performed within the Huttnehower lab’s galaxy instance using the relative abundance table (https://huttenhower.sph.harvard.edu/galaxy/; 93), with Kruskal-Wallis (P < 0.05), Pairwise Wilcoxon (P < 0.05), and logarithmic LDA score (>2.0). All included OTU analyses presented here were determined at a 97% sequence identity, allowing for genus-level resolution^32^, as determined using the furthest neighbor clustering algorithm. Correlational analysis was performed using Hmisc R package^33^.

### High-resolution metabolomics

Metabolomics sample processing was performed similar to that described by Mote, *et al*.^19^. Briefly, 50 μl of urine or plasma were mixed with 100 μl acetonitrile and 2.5 μl of internal standard, kept on ice (30 min) and centrifuged (10 min at 14,000 rpm). Metabolomics samples (100 μL of the supernatant) were analyzed on the Orbitrap Fusion Mass Spectrometer (Thermo Fisher), with instrument settings at 120,000 resolving power, 5 min runs, and 10 μl injection. Both reverse phase (negative mode) and hydrophilic liquid interaction chromatography (HILIC; positive mode) columns were used for each sample in triplicate. Peak detection, noise filtering, m/z and retention time alignment, feature quantification, and quality filtering were done using xMSanalyzer v2.0.7 with apLCMS v6.1.3^34,35^, running all samples (i.e., plasma and urine) simultaneously so any unique feature (i.e., unique *m/z* and retention time) identified would be the same regardless of biological matrix. Data were extracted as HRM features, and the average of three technical replicates were log_2_ transformed with a +1 pseudocount and quantile normalized prior to bioinformatics analysis.

### HRM data analysis

Feature intensity tables were analyzed in *R*. Circular Manhattan plots were generated using the CMplot R package^36^. PLS-DA were performed using the mixOmics R package v6.3.2., with near zero variance predictor filtering^30^. Pathway analysis was performed using *mummichog* within each date and for the entire grazing trial using the FDR corrected p-value and t-scores as input values, with respective ionization mode and [M + H] adduct matching^37^; all pathways presented herein are the −log10 mummichog corrected p-value.

### HRM and 16S rRNA gene data integration

For analyzing the interaction between the plasma/urine metabolomes and fecal microbiota, procrustes was performed on the Max-Q and E+ data independently (throughout the grazing trial and on Day 12 [−THI] and 20 [+THI]) with the vegan *R* package^38^, using the normalized 16S rRNA and combined plasma and urine metabolomes prior to running the monoMDS command to generate ordination plots. After running the *procrustes* function, the *protest* function was used to perform permutational Monte Carlo simulation to estimate the significance of the microbiota:metabolome correlation. Coinertia analysis (CIA) was performed on the Max-Q and E+ data independently (throughout the grazing trial and on Day 12 and 20) using the ade4 *R* package^39^ to estimate the covariance between the 16S rRNA OTUs and the plasma and urine metabolic features within a fescue cultivar. The CIA was done after combining the normalized plasma and urine metabolomes into one data frame prior to performing the *dudi*.*PCA* function. The *cia* function was used to perform the CIA with the OTU duality diagram as the X data matrix and the duality diagram of the HRM features as the Y data matrix. sPLS regression was done with the mixOmics *R* package v6.3.2^30^, using two components in the canonical mode with the plasma and urine combined HRM datasets, subset to each feature present in at least 50% of samples throughout the trial as the X matrix and the normalized 16S rRNA OTU table as the Y matrix. The Max-Q and E+ datasets were then separated and analyzed independently to assess relationships specific to fescue cultivar; correlation threshold was (|r| > 0.70). The resulting sPLS bipartite networks were saved in.gml format using the igraph *R* package^40^ for Cytoscape v3.6.0 visualization and differential network analysis.

### Statistical analysis of non-‘omics data

Statistical analyses of weight gains and urinary ergot alkaloids were done with Sigma Plot v12.5 (Systat Software, Inc., San Jose, CA) using two-way ANOVA (within-subjects design) with days of sampling and fescue treatment set as the two independent variables. If significant (*P* < 0.05) effects based on treatment or days spent grazing were observed, the Holm-Sidak *post-hoc* analysis was applied to separate significant differences. Graphs were generated with GraphPad Prism 5 (La Jolla, CA).

### Accession number(s)

All DNA sequences are publicly available in the NCBI Sequence Read Archive and are accessible under BioProject accession number PRJNA603062.

### Transparency document(s)

HRM feature intensity tables and metadata have been deposited in Metabolomics Workbench (www.metabolomicsworkbench.org).

## Results

### Environmental conditions, animal weight gains and urinary ergot alkaloids (UEAs)

THI values across sampling times were 69.9 (Day 0; Pre), 71.9 (Day 1), 70.8 (Day 2), 64.0 (Day 12; 1^st^ −THI), 74.3 (Day 16; 1^st^ +THI), 78.7 (Day 20; 2^nd^ +THI), and 74.0 (Day 26; 2^nd^ −THI); of note, rain on sampling Day 26 increased the relative humidity (used in THI calculation), but the temperature on this day was 23.64 °C, similar to day 2 (23.33 °C). E+ grazing steers had significantly (*P* < 0.05) lower average daily (0.5 ± 0.1 kg/day; ADG) and cumulative weight gains (13.9 ± 4.7 kg; CWG) compared to steers that grazed the non-toxic endophyte-infected tall fescue (Max-Q; [0.9 ± 0.3 kg/day ADG; 23.2 ± 6.9 kg CWG]) after the 26-day grazing period. Prior to pasture assignment (Day 0), the steers had some UEAs (67.9 ± 14.4 ng/mg creatinine), likely due to fescue hay supplementation. After pasture placement, UEAs in E+ animals were significantly (P < 0.001) increased beginning at 2 days throughout the remainder of the study (Day 2 = 221.3 ± 53.3, Day 12 = 246.9 ± 56.3, Day 16 = 296.9 ± 29.1, Day 20 = 234.4 ± 31.1, Day 26 = 253.8 ± 38.1 ng/mg creatinine), with Max-Q steers levels precipitously dropping and becoming markedly lower as the grazing trial progressed (Day 2 = 64.4 ± 37.9, Day 12 = 19.9 ± 10.1, Day 16 = 6.5 ± 4.8, Day 20 = 6.1 ± 4.2, Day 26 = 17.9 ± 6.9 ng/mg creatinine).

## Microbiota Results

### 16S rRNA gene sequencing

We generated 3,575,828 raw sequences, which resulted in 1,787,914 high quality sequences after filtering that clustered and aligned into 2,973 OTUs. The average number of paired sequences per sample was 21,285 (range: 7,035–60,852) and the average number of populated OTUs per sample was 954 (range: 493–1,298). The average Good’s coverage for the data set was 98.7 ± 0.6, indicating adequate sequencing depth and coverage to capture most of the species diversity in the samples.

### Alpha diversity metrics

Overall, both Chao1 richness and Simpson’s diversity had minor fluctuations throughout the trial. The highest diversity occurred between 2 and 16 days of grazing. Sample richness was slightly more variable, with the lowest richness being after 2 and 16 days and the highest richness being after 20 days of grazing. Neither Simpson’s diversity (P = 0.11) nor Chao1 richness (P = 0.35) were affected by fescue treatment in this study.

The overall fecal microbiota profile was significantly affected by fescue treatment and grazing time using both Jaccard (Treatment: P < 0.01; Time: P < 0.001) and Bray-Curtis (Treatment: P < 0.01; Time: P < 0.001) distance matrices. When comparing Day 12 (−THI) and Day 20 (+THI), there was a significant effect of treatment and a trend for an effect of THI for both Bray-Curtis (Treatment: P < 0.001; THI: P = 0.06) and Jaccard (Treatment: P < 0.001; THI: P = 0.14) distance matrices, without any significant effect on Inverse Simpson’s diversity or Chao1 richness measurements (P > 0.8).

### Microbial data reduction analysis

Partial least squares discriminant analysis (PLS-DA) showed that both Max-Q and E+ steer microbiota profiles shifted from the Pre steers in a distinct manner (Fig. S1A). Further, the samples later in the grazing period had shifted furthest from the Pre steers, with no significant specific shifts on −THI and +THI sampling dates. PLS-DA at each sampling date indicated the Max-Q and E+ steers formed distinct clusters, with the separation and clustering occurring along the first principal component (Fig. S1A). Generally, clustering along the second principal component was only seen in Max-Q steers on Day 2, 20, and 26 (Fig. S1A) and E+ on day 20 (Fig. S1A). Overall, the first component explained >90% of the overall variance, with >99% of the cumulative variance explained when the second component was added for each sampling date (Fig. S1A).

From the PLS-DA loadings analysis, classified families that overlapped between sampling dates and had OTUs as drivers of the Max-Q and E+ separation included those in the families *Erysipelotrichaceae*, *Lachnospiraceae*, *Ruminococcaceae*, *Rikenellaceae*, *Mogibacteriaceae*, *Coriobacteriaceae*, *Rikenellaceae* and *Clostridiaceae* (throughout the trial), the *Paraprevotellaceae*, *Peptostreptococcaceae*, candidate family *BS11* (early to midtrial), and candidate *S24-7* (late in the trial). A detailed list of all bacteria separated by sampling dates can be found in Table S1.

Day 12 (−THI) versus Day 20 (+THI) PLS-DA, irrespective of fescue treatment, revealed two distinct clusters, with Component 1 explaining 83.58% and Component 2 explaining 15.40% of the cumulative variance for the analysis (Fig. S1B). The top OTU loading weights were aligned to the family *Lachnospiraceae* (6 OTUs), with all other classified families (e.g., *Ruminococcaceae*, *Coriobacteriaceae*, and *Peptostreptococcaceae*) only having 1 OTU each.

Similar analyses were performed within E+ to assess the specific effects of THI on the E+ microbiota profile. When comparing Day 12 (−THI) versus Day 20 (+THI), the Day 12 and Day 20 steers formed distinct clusters, with the first component contributing 82.50% and the second component contributing 16.70% of the explained variance (Fig. S1B). *Ruminococcaceae* and *Lachnospiraceae* were the two most prevalently classified families that were driving the THI-based separation in E+ steers. Seventeen of the top 50 OTUs driving the separation were unclassified at the order level. *Mogibacteriaceae*, *Clostridiaceae*, *Coriobacteriaceae*, and *Porphyromonadaceae* were among other classified families responsible for the E+ THI-based separation.

### Linear discriminant analysis of effect size (LEfSe)

**Figure 1:**
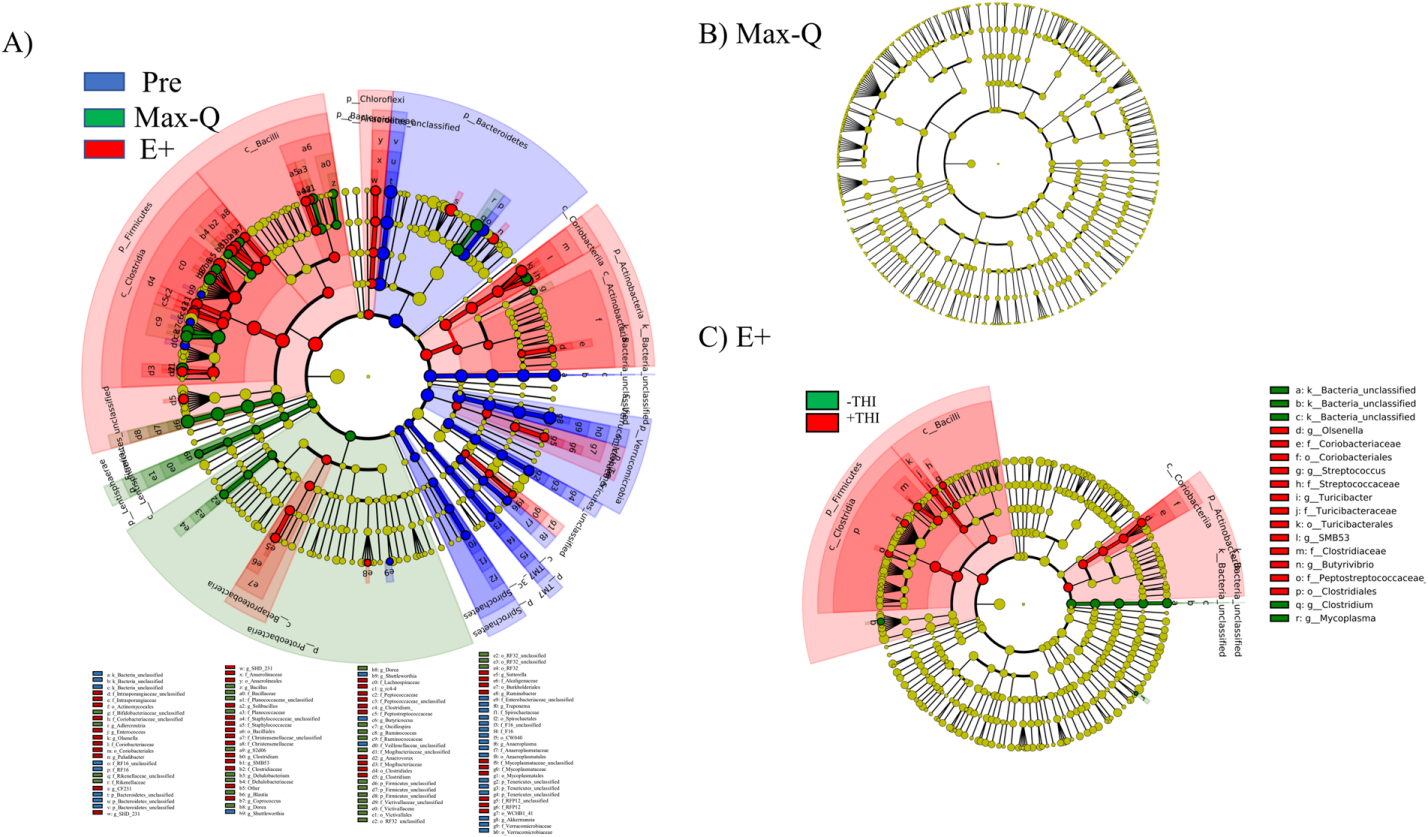
Linear discriminant analysis (LDA) effect size (LEfSe; Kruskall-Wallis [P < 0.05]; Pairwise Wilcoxon [P < 0.05]; logarithmic LDA score > 2.0) of the fecal microbiota of **(A)** Angus steers before (Pre) placement or across a 26-day grazing trial after placement on either a non-toxic (Max-Q; n = 6) or toxic (E+; n = 6) endophyte-infected tall fescue; **(B)** steers grazing Max-Q tall fescue on Day 12 (- temperature humidity index [−THI]) versus Day 20 (+THI); **(C)** steers grazing E+ tall fescue on Day 12 (−THI) versus Day 20 (+THI). **(A)** Blue, green, and red shading indicates greater abundance in Pre, Max-Q, or E+ steers, respectively **(C)** Green and red indicates greater abundance in E+ steers on Day 12 (−THI) and 20 (+THI), respectively. Taxonomic rank labels are provided before bacterial names: “p_; c_; o_; f_; g_” indicate phylum, class, order, family, and genus, respectively. Letters and numbers within the cladogram refer to bacterial names located in the keys below (panel A**)** and to the right (panel C) of the cladogram.

All effects listed as a result of the LEfSe met the following criteria for significance: Kruskal-Wallis P < 0.05; Pairwise Wilcoxon P < 0.05; logarithmic LDA score > 2.0. Overall, Max-Q grazing significantly increased the *Lentisphaerae* and *Proteobacteria* phyla (Fig. 1A). For the *Actinobacteria*, Max-Q grazing significantly increased the *Coriobacteriaceae* genus *Adlercreutzia*. From the class *Bacilli* within the phylum *Firmicutes*, Max-Q increased the *Planococcaceae* family and the *Bacillus* genus. Max-Q grazing also significantly increased the genus *Dehalobacterium*, the *Lachnospiraceae* genera *Dorea* and *Blautia*, and the *Ruminococcaceae* genera *Ruminococcus* and *Oscillospira* (Fig. 1A). Finally, within the *Bacteroidetes* phylum, the *Rikenellaceae* was significantly increased in Max-Q steers (Fig. 1A).

Overall, E+ grazing significantly increased the abundance of the *Firmicutes*, *Chloroflexi*, and *Actinobacteria* phyla. Within the *Proteobacteria*, the genera *Ruminobacter* and *Suttrella* were significantly increased in E+ steers (Fig. 1A). Further, E+ grazing significantly increased the candidate genus *SHD-231* (of the family *Anaerolinaceae* and phylum *Chloroflexi*; Fig. 1A). For the *Actinobacteria*, E+ grazing increased *Intrasporangiaceae* and the *Coriobacteriaceae* genera *Enterococcus* and *Olsenella* (Fig. 1A). The families *Mycoplasmataceae* and candidate *RFP12* of the *Verrucomicrobia* were significantly increased by E+ grazing. From the class *Bacilli* in the phylum *Firmicutes*, E+ grazing increased the genus *Solibacillus* of the family *Planococcaceae*. The *Clostridium* and candidate genera *SMB53* of the family *Clostridiaceae*, the genus *Coprococcus* in the family *Lachnospiraceae*, the candidate genus *rc4-4* of the family *Peptococcaceae*, and the genus *Anaerovorax* of the proposed family *Mogibacteriaceae* were all increased in E+ steers (Fig. 1A). Finally, within the *Bacteroidetes*, E+ grazing increased the genera *Paludibacter* and candidate *CF231* (Fig. 1A).

When comparing Day 12 (−THI) and Day 20 (+THI) within Max-Q steers, there were no significant effects on any fecal bacteria in response to the different THI conditions (Fig. 1B). However, when comparing the E+ steers, we found that the genera *Mycoplasma* and *Clostridium* of the family *Ruminococcaceae* were significantly decreased by +THI (Fig. 1C). Additionally, the *Coriobacteriaceae* genus *Olsenella*, *Streptococcaceae* genus *Streptococcus*, *Turicibacteraceae* genus *Turicibacter*, *Clostridiaceae* candidate genus *SMB53*, the genus *Butyrivibrio*, and the family *Peptostreptococcaceae* were all significantly increased in E+ steers under +THI conditions (Fig. 1C).

### Microbiota OTUs and THI/ADG correlation analysis

These correlational analyses of bacterial OTUs were performed to test if OTUs within individual families have either a positive or negative correlation with the endpoint of interest. Overall, the *Lachnospiraceae* and *Ruminococcaceae* families, alongside the proposed family *Mogibacteriaceae*, had OTUs whose relative abundances were positively correlated with ADG (r > 0.3; P < 0.05). Further, *Ruminococcaceae*, proposed *Paraprevotellaceae*, *Lachnospiraceae*, and *Bacteroidaceae* families had OTUs whose relative abundance negatively correlated with ADG. The genus *Prevotella* had one OTU that negatively correlated strongly with ADG (r = −0.68; P < 0.001). Within E+ steers, the classified families with the greatest number of negatively correlated OTUs were the *Ruminococcaceae*, *Lachnospiraceae*, *Bacteroidaceae*, *Mycoplasmataceae*, and *Clostridiaceae* (Table 1).

**Table 1:** Top classified families and genera correlating with average daily gains (ADG), urinary ergot alkaloids (UEAs) and temperature humidity index (THI) in steers grazing toxic (n = 6; E+) tall fescue for a 26-day grazing trial.

Family	Genus	Number of OTUs	Spearman’s R	P-value
**ADG (+r)**				
*Lachnospiraceae*		13	0.458	0.027
	*Dorea*	1	0.460	0.024
*Ruminococcaceae*		9	0.462	0.025
	*Oscillospira*	1	0.454	0.026
	*Ruminococcus*	1	0.426	0.038
*Bacteroidaceae*		3	0.499	0.023
	*5-7N15*	3	0.499	0.023
*Coriobacteriaceae*		3	0.471	0.028
*Erysipelotrichaceae*		2	0.473	0.026
	*p-75-a5*	1	0.412	0.045
**ADG (−r)**				
*Lachnospiraceae*		5	−0.494	0.016
	*Butyrivibrio*	2	−0.451	0.029
*Ruminococcaceae*		3	−0.440	0.033
	*Clostridium*	1	−0.406	0.049
	*Ruminococcus*	1	−0.480	0.018
**THI (+r)**				
*Lachnospiraceae*		24	0.417	0.019
	*Butyrivibrio*	3	0.394	0.018
	*Blautia*	1	0.441	0.007
	*Dorea*	1	0.550	0.001
*Ruminococcaceae*		12	0.395	0.023
	*Ruminococcus*	6	0.400	0.020
*Coriobacteriaceae*		9	0.417	0.019
	*Olsenella*	4	0.455	0.015
	*Enterococcus*	1	0.393	0.018
[*Mogibacteriaceae*]		7	0.446	0.016
	*Mogibacterium*	3	0.395	0.020
*Clostridiaceae*		3	0.453	0.011
	*Clostridium*	2	0.399	0.016
	*SMB53*	1	0.600	0.0003
**THI (−r)**				
*Ruminococcaceae*		15	−0.470	0.010
	*Oscillospira*	2	−0.386	0.020
	*Papillibacter*	1	−0.362	0.030
*Lachnospiraceae*		12	−0.423	0.016
	*Clostridium*	2	−0.425	0.009
[*Mogibacteriaceae*]		2	−0.382	0.024

The number of OTUs within a family/genus, average Spearman correlation coefficients (|r| > 0.30), and average P-values (P < 0.05) are presented.

When considering all steers, five classified families had OTUs with relative abundances positively correlating (r > 0.3; P < 0.001) with THI. Within E+ steers, the same classified families, excluding the *Peptostreptococcaceae* and including the *Ruminococcaceae*, positively correlated with THI (Table 1). Many OTUs were classified at the genus level as well, with *Olsenella* having the highest number of OTUs.

*Ruminococcaceae* was the only classified family that had OTUs with relative abundances negatively correlating (r < −0.3; P < 0.001) with THI, regardless of fescue treatment. Within E+, the top classified families that negatively correlated with THI were the *Ruminococcaceae*, *Lachnospiraceae*, *Erysipelotrichaceae*, *Mogibacteriaceae*, and candidate family *RFP12* (Table 1). For all analyses, numerous OTUs were classified at the genus level, and these genera are provided in Table 1.

### High-resolution metabolomics (HRM)

#### Descriptive statistics

A total of 12,030 and 12,407 HRM unique features were detected in the, respectively, plasma and urine with C18-LCMS; 16,878 and 17,484 HRM features were detected using HILIC-LCMS. Overall, a large number of HRM features were significantly influenced by E+ grazing throughout the grazing trial in both plasma and urine (Fig. S2). More specifically, a total of 1,753 (C18-LCMS) and 2,642 (HILIC-LCMS) HRM features were significantly (P < 0.05) affected by E+ grazing in the plasma, and 1,348 (C18-LCMS) and 3,250 (HILIC-LCMS) features were significantly (P < 0.05) affected by E+ grazing in the urine.

#### HRM data reduction analysis

HRM PLS-DA revealed results similar to the microbial analyses for both plasma and urine, in that both Max-Q and E+ steers metabolomes distinctly shifted from the metabolome composition prior to pasture placement (Pre; Fig. S3). Plasma PLS-DA loadings were metabolites primarily involved in Vitamin A (retinol), amino acid (e.g., tryptophan, tyrosine), butanoate, and arachidonic acid metabolism (throughout the trial), glutathione metabolism (early in the trial [Day 1–2]), biopterin and energy related metabolism (e.g., TCA cycle; late in the trial [Day 12–26]). The urine loadings were putative metabolites primarily involved in glycolysis and gluconeogenesis, amino acid, leukotriene and arachidonic acid, C21-steroid hormone metabolism, prostaglandin formation from arachidonate, and urea cycle/amino group metabolism (throughout the trial) and energy metabolism (e.g., fatty acid β-oxidation, etc.) and androgen/estrogen biosynthesis and metabolism (Day 16, 20, and 26).

When comparing Day 12 (−THI) and 20 (+THI) within E+ steers, we found that those HRM features that drove their separation were metabolites putatively involved in: prostaglandin formation from arachidonate, arachidonic acid metabolism, C21-steroid hormone biosynthesis and metabolism (plasma and urine) leukotriene and biopterin metabolism (plasma) and bile acid biosynthesis, aspartate and asparagine metabolism, arachidonic acid metabolism, non-unsaturated fatty acid beta-oxidation, and Vitamin E metabolism (urine).

#### HRM pathway analysis

**Figure 2:**
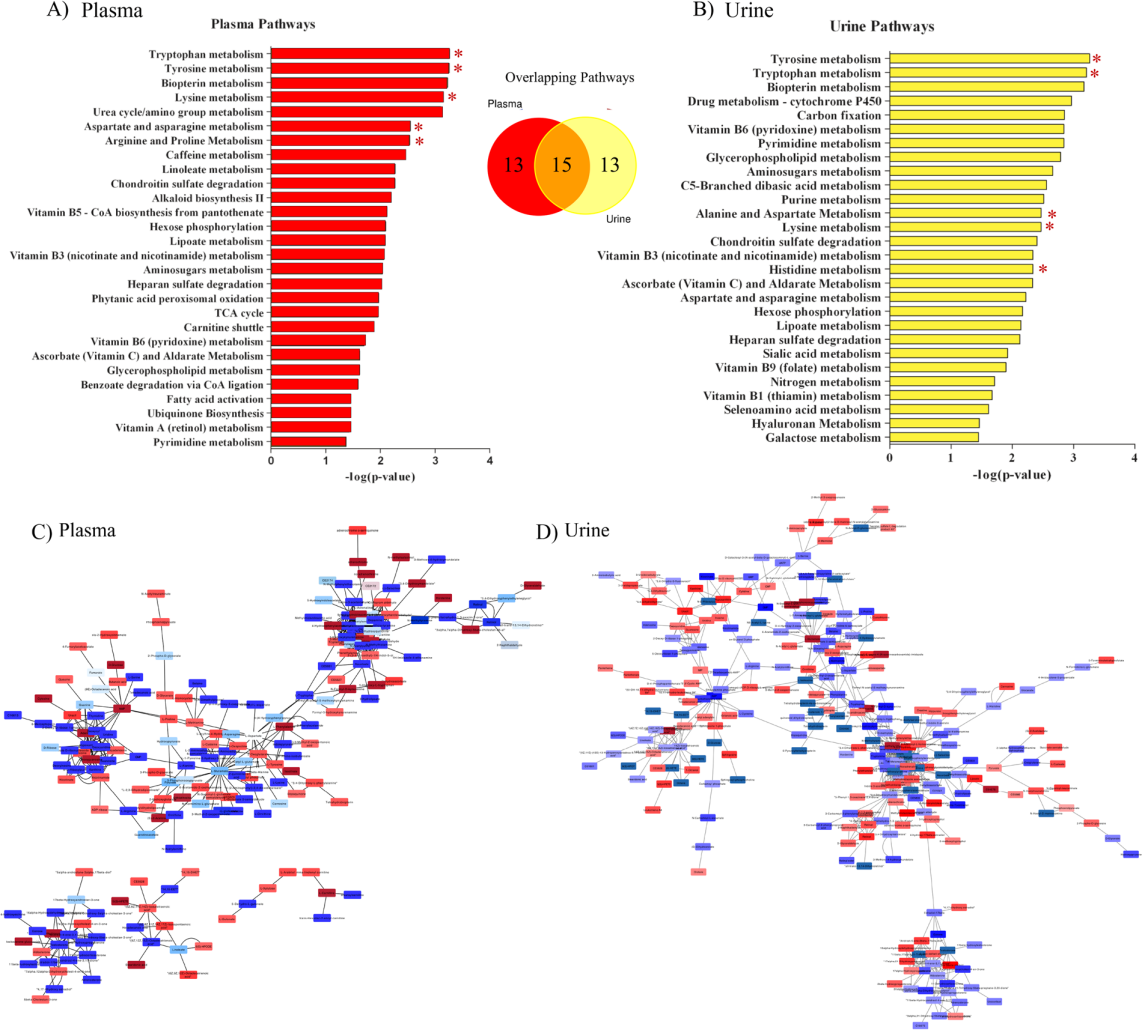
Pathway analysis performed on the **(A)** plasma (red) and **(B)** urine (yellow) high-resolution metabolomics features using the mummichog python program that indicates putative metabolic pathways significantly (P < 0.05) affected by toxic tall fescue (E+) grazing in beef steers throughout the 26-day grazing trial (n = 12). The x-axis indicates the negative log of the FDR corrected p-value for each metabolic pathway indicated on the y-axis. The Venn Diagram details the number of metabolic pathways that were significantly affected by E+ grazing in the plasma (red), urine (yellow), or in both (overlapping). Red asterisk indicates production (i.e., weight gain)-related metabolic pathways. Temporally merged activity networks for the (**C**) plasma and (**D**) urine resultant from the mummichog pathway analysis detailing specific putatively annotated metabolites that were either significantly increased (red) or decreased (blue) in steers grazing E+ (n = 6) tall fescue when compared to steers grazing a non-toxic tall fescue (n = 6) over the course of a 26 day grazing trial.

The overall top three pathways influenced by E+ grazing throughout the trial, for both plasma and urine, were tryptophan, tyrosine, and biopterin metabolism (Fig. 2A,B). Further, a number of amino acid metabolic pathways were significantly altered by E+ in both plasma and urine (Fig. 2A,B). Other significantly altered pathways in the plasma include lineolate metabolism and important energy producing pathways (e.g., TCA cycle and carnitine shuttle; Fig. 2A). In the urine, other metabolic pathways affected by E+ grazing include those involved in carbon fixation, drug, vitamin, lipid, and nucleic acid metabolism (Fig. 2B). Temporal merging of the mummichog activity networks resulted in clusters being formed around metabolites involved in amino acid and lipid metabolism, indicating these specific metabolic perturbations may be important for FT etiology (Fig. 2C,D).

**Figure 3:**
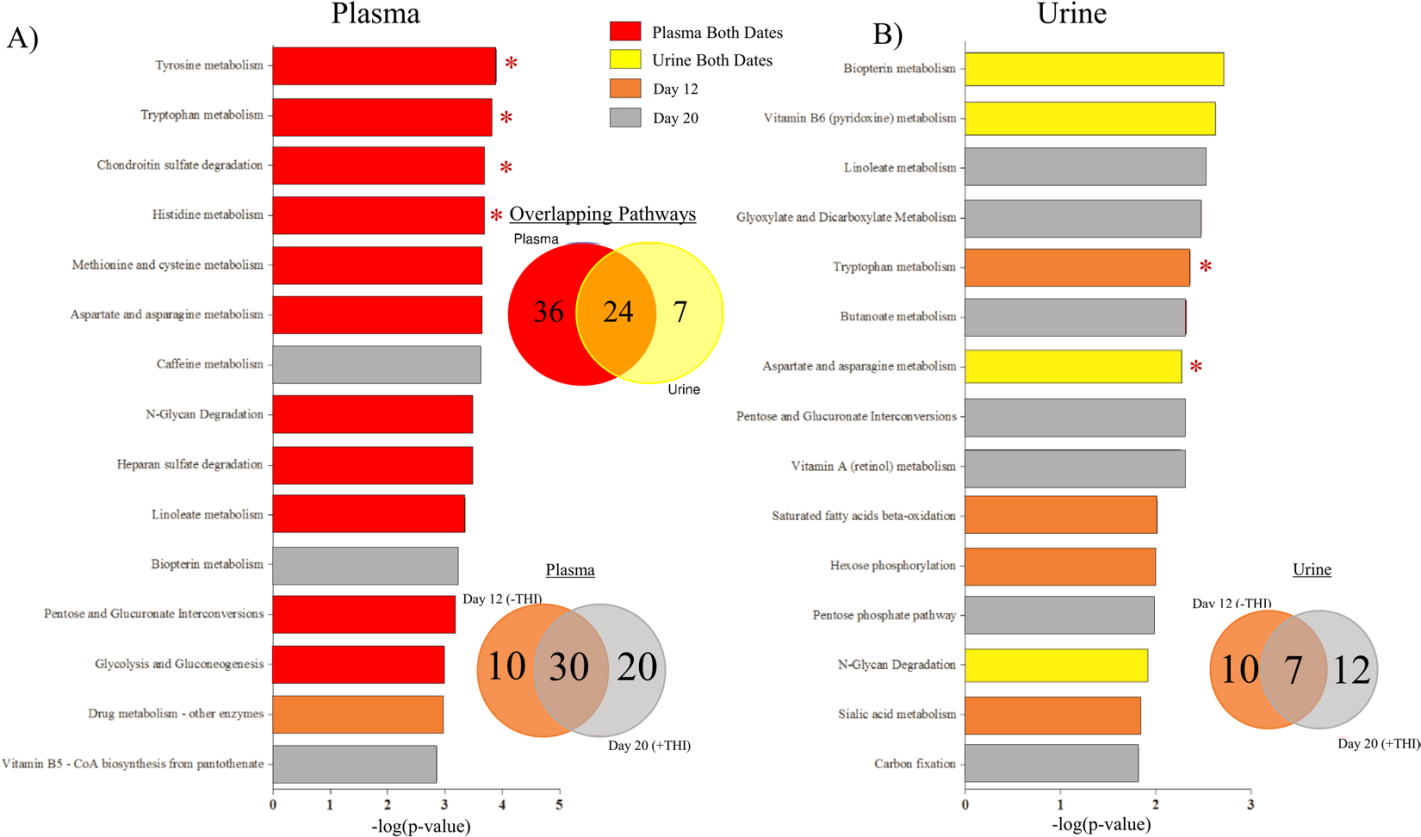
Top 15 pathways from pathway analysis performed on the **(A)** plasma and **(B)** urine high-resolution metabolomics features using the mummichog python program within Day 12 (−THI) and 20 (+THI), with putative pathways that are significantly affected by toxic tall fescue (E+) grazing solely on Day 12 (−THI; orange), only on Day 20 (+THI; gray), or on both dates (plasma = red; urine = yellow). The Venn diagrams detail the number of putative metabolic pathways that were affected in the plasma and/or urine (top) or in the plasma on Day 12 and/or Day 20 and (bottom left) or in the urine on Day 12 and/or Day 20 (bottom right).

There were a number of metabolic pathways that were significantly affected by E+ grazing on least (−THI) and most (+THI) harsh days of the grazing trial (Fig. 3). Overall, 24 metabolic pathways were significantly affected in both plasma and urine, whereas 36 and 7 were specifically affected in, respectively, the plasma or the urine (Fig. 3). Within the plasma, 30 of the resultant metabolic pathways were THI-independent, and 10 and 20 metabolic pathways were affected on Day 12 or Day 20, respectively (Fig. 3). In the urine, only 7 metabolic pathways were significantly affected by E+ grazing independent of THI, with 10 and 12 being specific to Day 12 and 20, respectively (Fig. 3). These data indicate that more metabolic pathways in the plasma than in the urine are affected by E+ and that the +THI conditions modify this effect.

More specifically, in the plasma, tyrosine and tryptophan were the top two pathways significantly affected by E+ grazing, regardless of THI. Other highly affected metabolic pathways include chondroitin sulfate degradation, histidine metabolism, methionine and cysteine metabolism and aspartate and asparagine metabolism (Fig. 3). In the urine, the top altered metabolic pathways were biopterin and Vitamin B6 metabolism on both Day 12 and Day 20 (Fig. 3). Detailed list of metabolic pathways affected irrespective of THI or on either Day 12 or 20 can be seen in Fig. 3.

#### Plasma and urine sPLS networks

For both Max-Q and E+ steers, sPLS variable selection resulted in two distinct clusters, one with plasma metabolites (orange) as the anchors and one where plasma and urine metabolites are dispersed evenly throughout the network (Fig. S4). The Max-Q Day 12 (−THI) and 20 (+THI) networks were similar to those of the overall network. On the other hand, while the E+ Day 12 was similar, the E+ Day 20 network had urinary metabolites as the anchors of both clusters within the network (Fig. S4). In support, the metabolites involved in the Max-Q networks were putatively identified as involved in methionine and cysteine, tyrosine, and arginine and proline metabolism, regardless of THI. While the E+ metabolites in the overall and Day 12 networks were similar, the Day 20 E+ network had metabolites involved in multiple pathways only existent on Day 20 (e.g., pentose phosphate pathway, C21-steroid hormone biosynthesis and metabolism, and tryptophan, tyrosine, and xenobiotics metabolism) (Fig. S4).

#### Microbiota:metabolome integration

**Figure 4:**
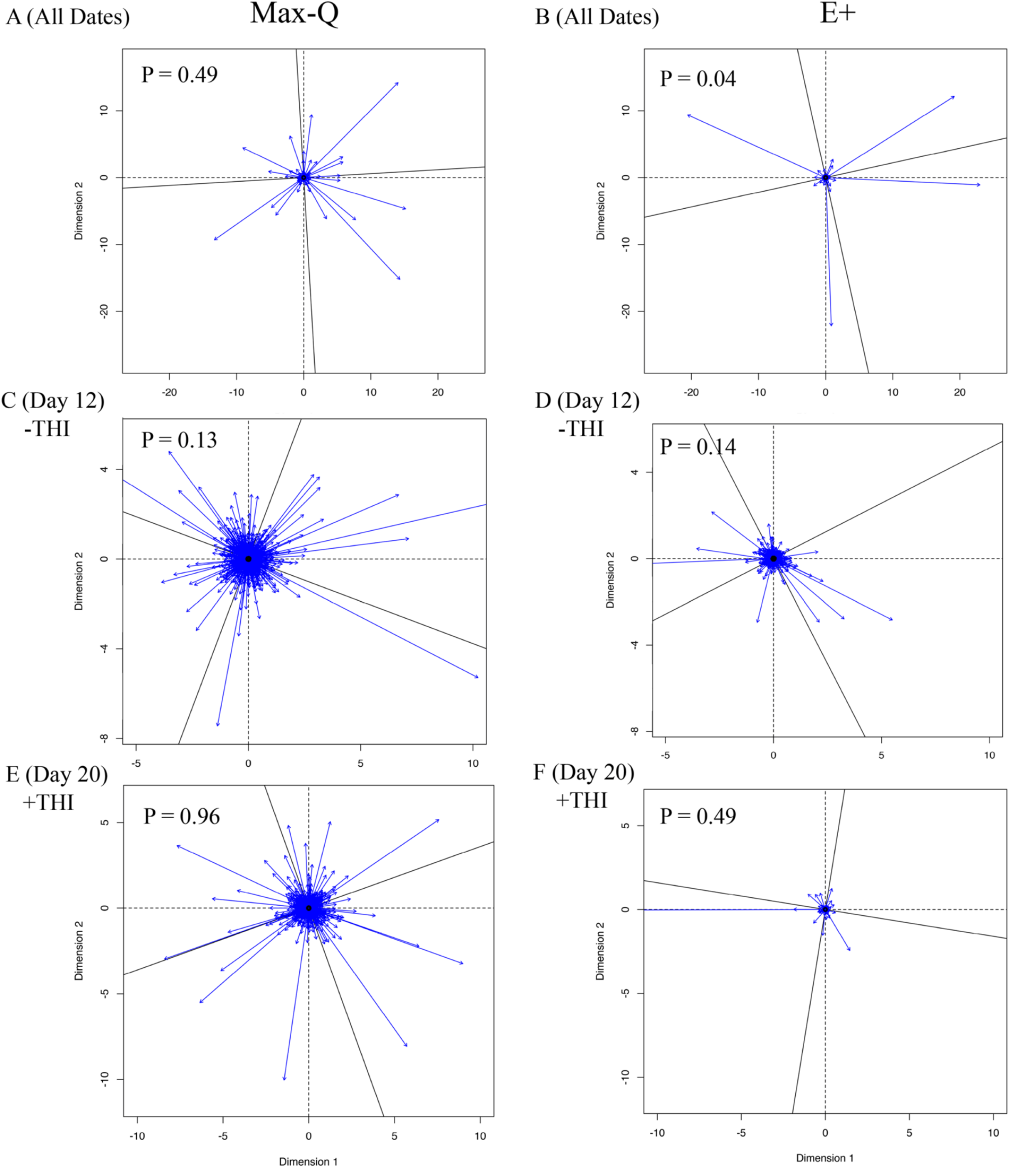
Procrustes analysis performed using the *vegan* R package for steers grazing either a (**A**; Max-Q; n = 6) novel, non-toxic or (**B**; E+; n = 6) toxic endophyte-infected tall fescue over the course of a 26-day grazing trial; Max-Q steers on Day 12 (**C**; −THI) and Day 20 (**E**; +THI) and E+ steers on Day 12 (**D**; −THI) and Day 20 (**F**; +THI). The significance values were estimated using the *protest* function to perform permutational Procrustes, with the P values reported in the upper left hand side of each respective plot. The greater vector length is indicative of greater inter-‘omic variability.

Procrustes analysis revealed that the overall E+ fecal microbiota and the total metabolome are overall strongly correlated (Fig. 4A, Monte Carlo P = 0.04); but this is not the case for Max-Q steers (Fig. 4B, Monte Carlo P = 0.49). The inter-‘omic relationship was not apparent when the procrustes analyses was done on a subset of the data within −THI (Fig. 4C,D) or +THI (Fig. 4E,F). We found that the Monte Carlo simulation insignificance was similar for Max-Q (P = 0.13) and E+ (P = 0.14) steers under thermoneutral conditions (Day 12) or during +THI (i.e., Day 20), although the E+ (P = 0.49) correlation was numerically stronger than the Max-Q one (P = 0.96).

**Figure 5:**
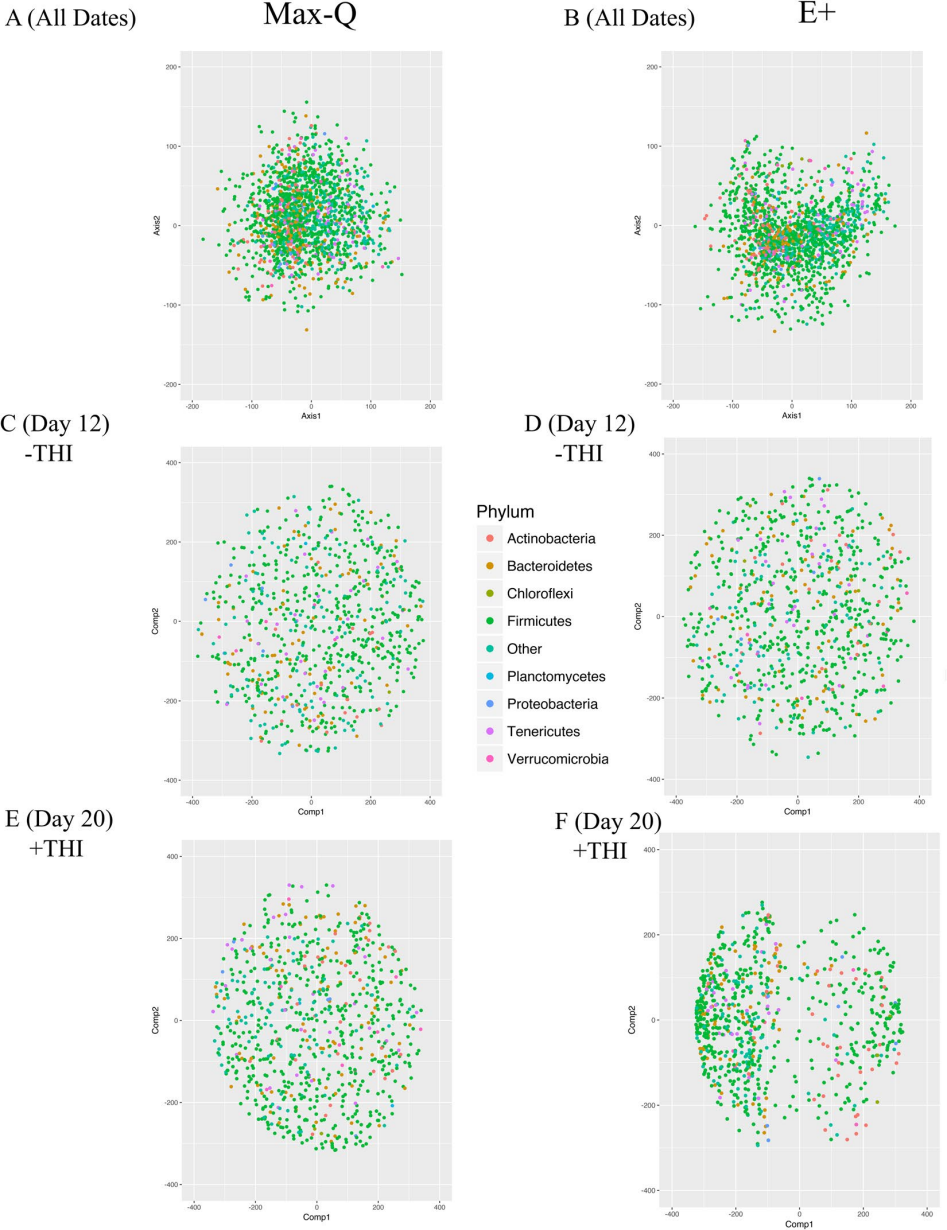
Coinertia analysis performed on OTUs for steers grazing either a (**A**; Max-Q; n = 6) novel, non-toxic or (**B**; E+; n = 6) toxic endophyte-infected tall fescue over the course of a 26-day grazing trial; Max-Q steers on Day 12 (**C**; lowest −THI) and Day 20 (**E**; highest +THI) and E+ steers on Day 12 (**D**; −THI) and Day 20 (**F**; +THI). Each point is plotted based on the OTUs predicted covariance with the plasma and urine metabolomes (i.e., greater distance from the point of origin indicates a greater covariance) of the respective group of steers. Red = *Actinobacteria*, brown = *Bacteroidetes*, green = *Firmicutes*, teal = other (unclassified, *Planctomycetes*, *Chloroflexi*, and other low abundance phyla), blue = *Proteobacteria*, purple = *Tenericutes*, and pink = *Verrucomicrobia*.

Coinertia analysis (CIA) indicated that OTUs classified to the *Firmicutes* had strong inter-omic covariance in both Max-Q and E+ steers, but the magnitude of this covariance is higher in E+ steers (Fig. 5A,B). Further, the covariance (i.e., distance from the origin) for a number of *Actinobacteria*, *Tenericutes*, and *Verrucomicrobia* OTUs was higher in E+ steers (Fig. 5A,B). Finally, a number of low-level phyla (i.e., <10 OTUs; labeled as Other) had high covariance in E+ steers but not in Max-Q steers (Fig. 5A,B). Under thermoneutral conditions (Day 12), no significant shift between the microbiota-total metabolome covariance was observed between Max-Q (Fig. 5C) and E+ (Fig. 5D) steers. On Day 20, the Max-Q (Fig. 5E) CIA revealed a similar pattern to Max-Q Day 12, but the E+ (Fig. 5E) CIA revealed a distinct shift in the microbiota-metabolome covariance leading to a bi-modal distribution of OTUs. Notably, the magnitude of *Firmicutes* OTUs covariance was greater, but this similar differential pattern was similar for almost all phyla, i.e., most OTUs had significantly shifted from the point of origin. This indicates that the overall E+ effect on CIA was driven by greater differences during +THI.

**Figure 6:**
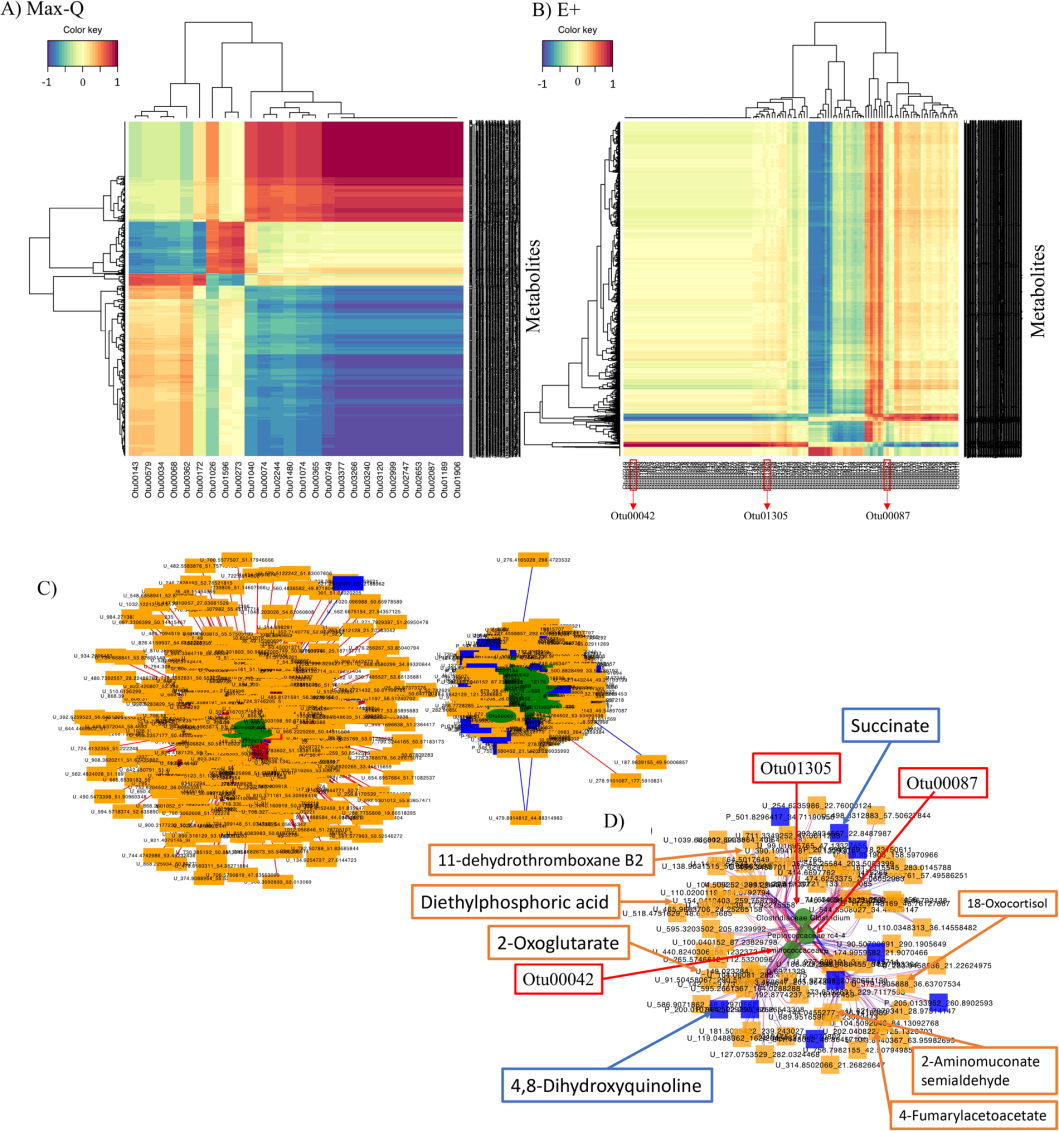
Heat maps representing fecal 16S rRNA OTUs and plasma high-resolution metabolomics (HRM) features that were selected by sparse partial least squares regression (sPLS) and high correlation (|r| > 0.7) in steers grazing either a novel, non-toxic (**A**; Max-Q; n = 6) or toxic (**B**; E+; n = 6) tall fescue over the course of a 26 day grazing trial. Red and blue indicates positive and negative correlations, respectively. Select OTUs proposed as potential E+ -associated microbiota biomarkers are boxed in red on the x-axis of panel B. Bipartite network of fecal 16S rRNA OTUs (green circles) and plasma (orange rectangles) and urine (blue rectangles) high-resolution metabolomics (HRM) features that were selected by sparse partial least squares regression (sPLS; top 100 OTUs [X matrix] and 500 plasma and urine HRM features [Y matrix]) using the *mixOmics* R package and were highly correlated (|r| > 0.7) **(C)** throughout the grazing trial and **(D)** a subnetwork of selected OTUs (green circles) that correlated with average daily weight gains, urinary ergot alkaloids (biomarkers of exposure and key etiological agents of FT), and THI, with the plasma (blue rectangles) and urine (red rectangles) HRM features that were highly correlated (|r| > 0.7) with these OTUs in the original network. Red and blue edge indicates positive or negative correlations, respectively. Select OTUs proposed as potential E+ -associated microbiota biomarkers pointed to by red arrows as the anchors of the network in panel D. Select plasma (blue box and arrows) and urine (orange box and arrows) metabolic features identified in the subnetwork have been highlighted in panel D.

After sPLS variable selection using the data from all dates post pasture placement, Max-Q steers had significantly fewer OTUs and metabolomics features that had strong correlation (|r| > 0.7), supporting the results of the Procrustes and CIA analyses (Fig. 6A,B). Considering this, differential network analysis was performed. In the resultant E+ network, most of the selected HRM features that were highly correlated with fecal OTUs were urinary metabolites (orange; 486 features; Fig. 6C), but plasma metabolites were also part of the network (blue; 30 features; Fig. 6C). Further, two distinct clusters were formed and most of the plasma HRM features were in the cluster on the right; most urine HRM features were in the left cluster (Fig. 6C). In the left cluster, the OTUs that correlated with urinary metabolites were mainly aligned in the *Ruminococcaceae* and *Lachnospiraceae*, with one OTU aligned in the candidate family *S24-7* and one unclassified at the family level (Fig. 6C). The plasma metabolites in the left subnetwork mapped to C21-steroid hormone biosynthesis and metabolism, and tryptophan and tyrosine metabolism (Fig. 6C). Similarly, in the right subnetwork, most OTUs also aligned to the *Ruminococcaceae*, *Lachnospiraceae*, and *Erysipelotrichaceae* families, with OTUs also aligning in the candidate *RFP12* and *Mogibacteriaceae* families (Fig. 6C). The plasma and urinary metabolites were involved in tryptophan, tyrosine, and androgen and estrogen biosynthetic metabolic pathways (Fig. 6C). Finally, many of the urinary and plasma HRM features were unidentified, and many OTUs were unclassified at the family level.

Interestingly, one OTU (Otu00087) aligned to *Peptococcaceae* candidate genus *rc4-4* within the sPLS network and was also significantly correlated (+r) with ADG and UEAs (−r) in E+ steers. Another OTU (Otu01305) aligned to the *Clostridiaceae* genus *Clostridium* and correlated with both ADG in E+ steers and THI (+r). Finally, a *Ruminococcaceae* OTU (Otu00042) correlated with THI and UEAs in E+ steers (+r). These three OTUs shared most of the highly significantly correlated plasma and urinary metabolites which were involved in tyrosine and tryptophan metabolism, valine, leucine and isoleucine degradation, and C21- steroid hormone biosynthesis. Otu01305 was also peripherally connected to metabolites involved in prostaglandin formation from arachidonate (Fig. 6D). Notably, these three OTUs were present when sPLS and differential network analysis was performed on Day 12 and Day 20 independently. So, although the abundance of these OTUs may be influenced by environmental conditions, as indicated by positive or negative THI correlations, these OTUs may also be robust predictors of E+ effects on the fecal microbiota:metabolome interactions regardless of environmental conditions.

## Discussion

The data presented herein provide important insights into the nature of the pathophysiological shifts that occur while beef cattle graze E+ tall fescue pastures and highlight the complexity of developing FT therapeutics, as both grazing and external (e.g., environmental) stressors influence the animal response to E+. In a 26-day grazing trial, we found that even relatively short-term grazing results in significant production deficits (i.e., significantly lower ADG) and rapid fluctuations in ambient environmental conditions modulate pathophysiological responses to E+ grazing. Although future evaluation of the ruminal microbiota, in addition to the fecal one as done here, to better understand FT development from a systemic level will be important moving forward, the results from the current study still have significant implications for future E+ therapeutic development and grazing management strategies that seek to help alleviate the economic and environmental burden of FT. As temperatures continue to rise, improving animal health and productivity for beef cattle grazing the predominant Southeastern U.S. pasture grass under environmentally stressful conditions is of great urgency.

We recently reported that E+ grazing significantly alters the fecal microbiota in grazing beef steers under thermoneutral conditions^18^. The data presented herein support the previous study, indicating Max-Q or E+ grazing results in rapid, endophyte-specific shifts in the fecal microbiota that persist throughout the grazing trial. In both studies, *Firmicutes* were the most prominently affected phylum by E+ grazing; however, here, we also found that E+ grazing results in a microbiota more sensitive to fluctuating environmental conditions. In this regard, it has already been shown that heat stress can influence the fecal microbiota in dairy cattle^41^. Recent evidence directly attributed heat stress to impaired bovine gut integrity and changes in the gut immune profile^42^. It has also been suggested that these changes in gut permeability and immune status can influence enteric microbiota populations^43,44^. In our study, there were numerous bacteria that were increased in E+ steers under +THI. However, the number of *Ruminococcaceae* OTUs that were significantly increased by E+ grazing was lower under +THI conditions, indicating that E+ -related *Ruminococcaceae* increases in the feces might be offset by harsh environmental conditions. Overall, these data indicate that the beef fecal microbiota is inherently dynamic, and it responds rapidly to both dietary and environmental exposures. While future studies assessing the ecological effects of E+ should further track ruminal and fecal OTUs associated with susceptibility/resistance to E+ production deficits and the environment, it may also be important to perform whole genome sequencing of the microbiome to assess whether there are characterized or novel bovine-specific small proteins that could have important regulatory or housekeeping functions for the microbiome, as was recently discovered for humans^45^.

HRM revealed that the most significantly affected metabolic pathways were the tryptophan, tyrosine, and biopterin metabolism in both the plasma and urine, supporting our previous metabolomics study with the more robust data being herein^19^. When we integrated the activity networks from sequential sampling dates, we found a highly interconnected network of metabolites anchored around amino acid metabolism. It is also noteworthy that metabolomics analyses were able to provide crucial insights into potential indirect effects on global metabolism resultant from ergot alkaloid and/or E+ exposure. Alkaloid biosynthesis II was one pathway significantly affected by E+ in the plasma. Of note, this pathway is not directly responsible for ergot or indole-alkaloid biosynthesis; however, through alterations in amino acid metabolism, it is likely the alkaloid biosynthetic pathway intersects with early steps of ergot alkaloid metabolism. Further, tryptophan and tyrosine metabolism, among other amino acids, are involved in numerous physiological processes, including skeletal muscle anabolism. For example, tryptophan has been previously shown to stimulate the IGF-1/mTORC1/S6K1 skeletal muscle building pathway in mice^46^, with previous studies finding E+ tall fescue can reduce serum IGF-1 concentrations^47,48^. It is well known that proteins/amino acids are important for skeletal muscle accretion in growing cattle (i.e., lean growth)^49^. Of note, recent investigations are beginning to elucidate the mechanisms by which amino acids regulate skeletal muscle autophagy in response to stressors, i.e. one study reported that amino acid supplementation can reduce muscle loss (autophagic) in neonatal pigs during endotoxemia^50^, indicating that alterations in amino acid metabolism as a result of E+ grazing could be related to decreased lean weight gain not only by decreasing skeletal muscle accretion but also by promoting skeletal muscle autophagy.

The data also showed that environmental conditions can modulate certain metabolic pathways that are significantly affected by E+ grazing. sPLS network visualization of the plasma and urine metabolomes revealed that the metabolic features that best describe the variability in these physiologically important data matrices were only sensitive to −THI and +THI fluctuations in E+ steers, indicating that the E+ steer metabolomes are more susceptible to environmental stressors than non-toxic fescue (i.e., Max-Q) grazing steers. Notably, PLS-DA loadings analysis revealed that, in E+ steers, the main metabolites driving the separation between −THI and +THI conditions were components of inflammatory metabolic pathways (e.g., arachidonic acid metabolism and prostaglandin formation). Previous studies found that moderate heat stress induces an immune/inflammatory response in dairy cattle^51,52^. As it relates to FT, steers on toxic tall fescue had greater serum TNF-α and cortisol levels than steers on endophyte-free tall fescue in response to LPS challenge^47^. Under stressful conditions, inflammation is just one factor contributing to alterations in global metabolism, and the animal will reprioritize important muscle building nutrients to meet allostatic load demand, ultimately resulting in decreased muscle accretion^53^. These data, coupled with impaired thermoregulation in E+ animals, indicate that under short-term E+ grazing, which is relevant to rotational grazing practices common to E+^54^, mild to moderate heat stress conditions could induce inflammatory responses, resulting in perturbed metabolism and increased nutrient demand to maintain thermoneutrality.

A noteworthy finding from the integrative analyses was that the non-toxic (Max-Q) steer microbiota had little predicted covariance with the plasma and urine metabolomes, but the fecal microbiota of E+ grazing steers significantly covaried with the metabolomes overall. Moreover, a number of urinary and plasma metabolites were significantly correlated with fecal OTUs and were involved in androgen/estrogen biosynthesis, C21-steroid hormone biosynthesis/metabolism, and fatty acid activation. As it relates to FT and animal productivity, a recent study has shown that androgen receptor signaling can influence myogenic differentiation through Wnt and TGF-β/Smad signaling^55^, potentially indicating that E+ grazing could result in perturbed muscle accretion by altering the microbiota-metabolome relationship. Also, the higher correlation and covariance of the E+ grazing steers, notably under +THI conditions, suggests decreased complexity of the host-microbe relationship. The microbiota:metabolome differences between Max-Q and E+ steers could be a result of changes in E+ gut microbial metabolism leading possibly to increased gut permeability^56^ or E+ effects resulting in a heat stress-susceptible physiological and/or immunological background prior to +THI exposure. These data point to potential biomarkers, either in plasma or urine, that could be readily accessed and used to identify the presence of particular bacteria associated with animal productivity in E+ grazing steers. As shown for necrotizing enterocolitis in humans^57^, these urinary biomarkers may be combined with other clinical signs (e.g., UEAs) to predict microbiota associated with low productivity, which is amenable to either dietary or therapeutic interventions.

We identified three OTUs that were unique to the E+ sPLS networks and were significantly associated with THI, UEAs, and ADG. These were *Peptococcaceae* candidate genus *rc4-4*, *Clostridiaceae* genus *Clostridium*, and one *Ruminococcaceae* OTU. *Peptococcaceae rc4-4*, which was significantly increased by E+, has been associated with increased thermogenesis in cold environments in mice^58^ and was found to be negatively associated with circulating amino acids and choline compounds and positively associated with circulating cholesterol and fatty acids^59^. Although it is unlikely the sole factor, this particular bacteria could potentially contribute to altered amino acid metabolism we have found here and under thermoneutral conditions^19^. The *Ruminococcaceae* and *Clostridiaceae* families are bacteria we have previously found affected by E+ grazing, and deep sequencing of these two families would help delineate which specific species/strains of bacteria may be most relevant for FT etiology. Nonetheless, these OTUs, or perhaps more easily the urinary metabolites associated with these OTUs, can be further assessed for their utility to detect E+ exposure related microbiota presence that is indicative of decreased animal production efficiency regardless of environmental conditions.

The only study, to our knowledge, directly assessing the effects of E+ grazing on methane production found no difference in methane levels for steers grazing endophyte-free and endophyte-infected tall fescue pastures^60^. Although we did not directly measure archaea, hydrogen-producing bacteria, such as the cellulolytic *Ruminococcous*, are important for methanogens and other bacteria have been associated with low- or high-methane producing microbiota (e.g., other *Ruminococcaceae*, *Lachnospiraceae*, *Prevotella*, etc. for high-producers^61^). While utilizing fecal bacteria as easily accessible biomarkers of ruminal methane production has previously been proposed^62^, the data herein suggest the urinary metabolome could be indicative of specific endpoints of the fecal microbiota, such as E+ or ruminal methane production. In addition to possible dyshomeostasis of methane production-related bacteria by E+, the decreases in animal productivity resultant from E+ grazing will inherently lead to increased herd size to meet pasture-based agricultural demands^1^; therefore, E+ grazing will, at the least indirectly, result in a greater contribution of E+ grazing animals to methane emissions. This phenomenon has to be studied in detail in the future. Ultimately, multiple studies, like the one performed herein, are beginning to highlight the utility of top-down strategic approaches^63^ for improving animal health and welfare.

## Conclusions

**Figure 7:**
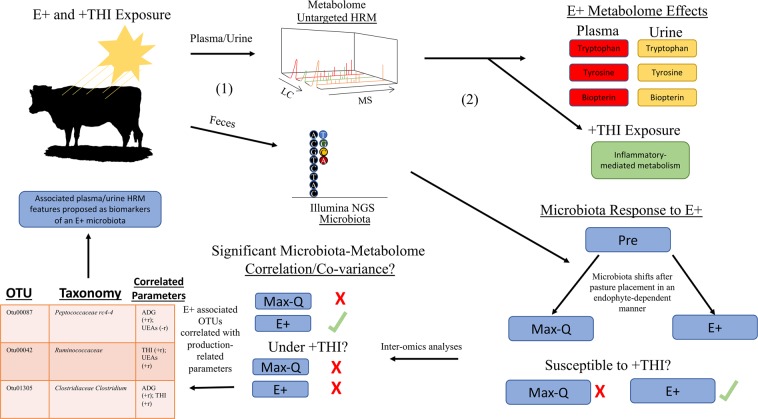
Summary figure representing the study design and major results. Angus steers (n = 12) were allowed to graze either a toxic (E+; n = 6) or non-toxic (Max-Q; n = 6) tall fescue pastures throughout a 26-day grazing trial. Samples were collected on dates where there was no risk of heat stress (−THI) and where there was a mild-to-moderate risk of heat stress (+THI). Plasma and urine samples were analyzed by high-resolution metabolomics (HRM) and fecal samples were subjected to next-generation sequencing techniques targeting the 16S rRNA gene. With bioinformatics, we found significant perturbations of both the metabolome and the microbiota, which were modulated by +THI conditions only in E+ steers. Further, only E+ steers in thermoneutral conditions had a significant correlation/covariance between the microbiota and metabolome and sparse partial least squares regression revealed three OTUs that were predictive of E+, regardless of environmental conditions and were highly correlated with plasma/urine metabolites. We propose the further exploration of these as biomarkers of an E+ microbiota associated with production deficits that could possibly be targeted therapeutically.

Overall, these data demonstrate that E+ grazing contributes to decreases in animal productivity through significant alterations in the microbiota and global metabolism. Further, E+ grazing results in greater susceptibility to environmental stressors such as increased heat and humidity, which are common in the Southeastern US and will become a greater burden on animal health and productivity as the climate continues to warm. As shown by our summary figure, E+ grazing resulted in significant metabolic and microbiota perturbations, while also increasing the susceptibility to +THI conditions (Fig. 7). As a result of this work, we were able to use bioinformatics techniques to identify important microbiota-metabolome relationships that could provide important, easily-accessible biomarkers for beef producers (Fig. 7). The increased susceptibility of E+ steers to +THI conditions indicate that, even for rotational grazing production settings, accounting for both environmental and seasonal changes will be important for developing efficacious microbial and/or metabolic-targeted FT interventions in the future. The data herein suggest that E+ grazing results in significant shifts in amino acid metabolism in the plasma, which could contribute to decreased skeletal muscle accretion by altering available amino acids or perturbing basal levels of skeletal muscle autophagy. Finally, the alterations in the microbiota-metabolome interaction could be used as biomarkers and as targets for management and/or therapeutic interventions.

## Supplementary information

Supplemental Data.
