# Supplementary material for: Toxic tall fescue grazing increases susceptibility of the Angus steer fecal microbiota and plasma/urine metabolome to environmental effects
Source: Sci Rep. 2020 Feb 12;10:2497. doi: 10.1038/s41598-020-59104-1 (PMC7016188; doi:10.1038/s41598-020-59104-1)
Supplement: Supplementary file 1 — Supplemental Data. [file 41598_2020_59104_MOESM1_ESM.pdf]

Toxic tall fescue grazing increases susceptibility of the Angus steer fecal microbiota and plasma/urine metabolome to environmental effects

Ryan S. Mote[1,2], Nicholas S. Hill[3], Joseph H. Skarlupka[4], ViLinh T. Tran[5], Douglas I. Walker[5], Zachary B. Turner[2], Zachary P. Sanders[3], Dean P. Jones[5], Garret Suen[4], Nikolay M. Filipov[1,2*]

[1] Interdisciplinary Toxicology Program,[2] Department of Physiology and Pharmacology,
[3] Department of Crop and Soil Sciences, University of Georgia, Athens, GA;
[4] Department of Bacteriology, University of Wisconsin – Madison, Madison, WI;
[5] Department of Medicine, Division of Pulmonary, Allergy, and Critical Care Medicine, Emory University, Atlanta, GA.

Supplemental Figures.

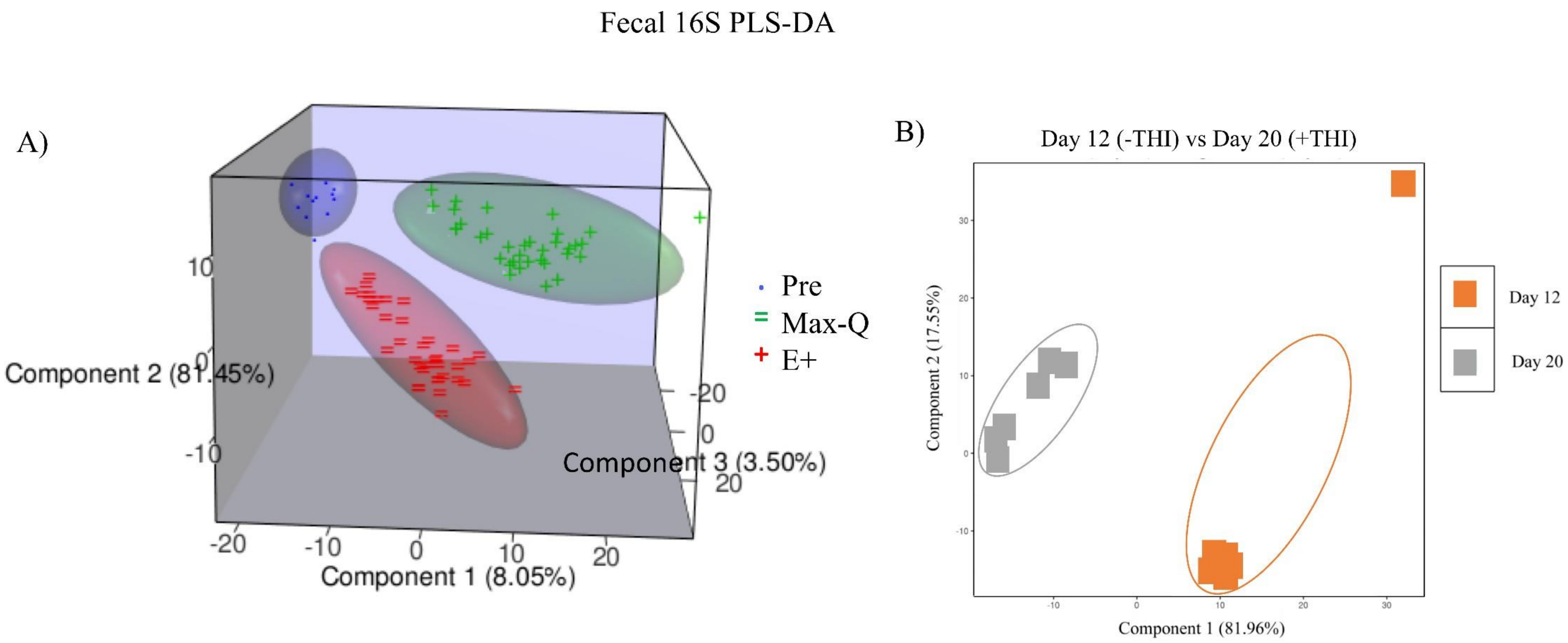

Fig. S1. Partial least squares discriminant analysis (PLS-DA) plots analyzing the fecal microbiota of Angus steers before pasture placement (blue) or grazing either a novel, non-toxic (Max-Q; n = 6; green) or toxic (E+; n = 6; red) endophyte-infected tall fescue (A) over the course of a 26-day grazing trial or (B) E+ grazing steers on Day 12 (-THI; orange) or Day 20 (+THI; gray) post pasture assignment.

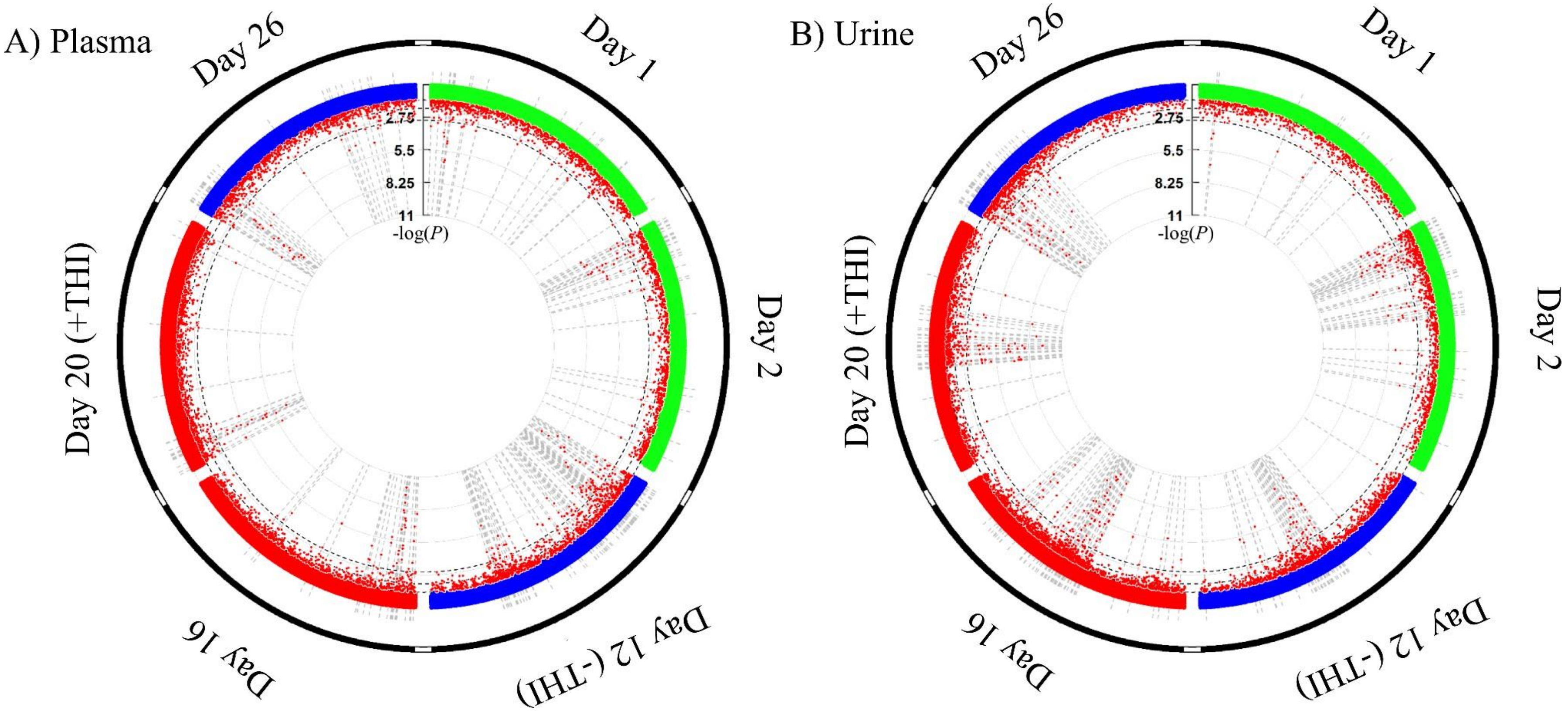

Fig. S2. Circular Manhattan plots representing high-resolution metabolomics (HRM) features in the (A) plasma and (B) urine of Angus steers grazing either a novel, non-toxic (Max-Q; n = 6) or toxic (E+; n = 6) endophyte-infected tall fescue over the course of a 26 day grazing trial. Red dots indicates HRM features were significantly affected by E+ treatment (FDR corrected P < 0.05) and the more central the dots the greater the significance.

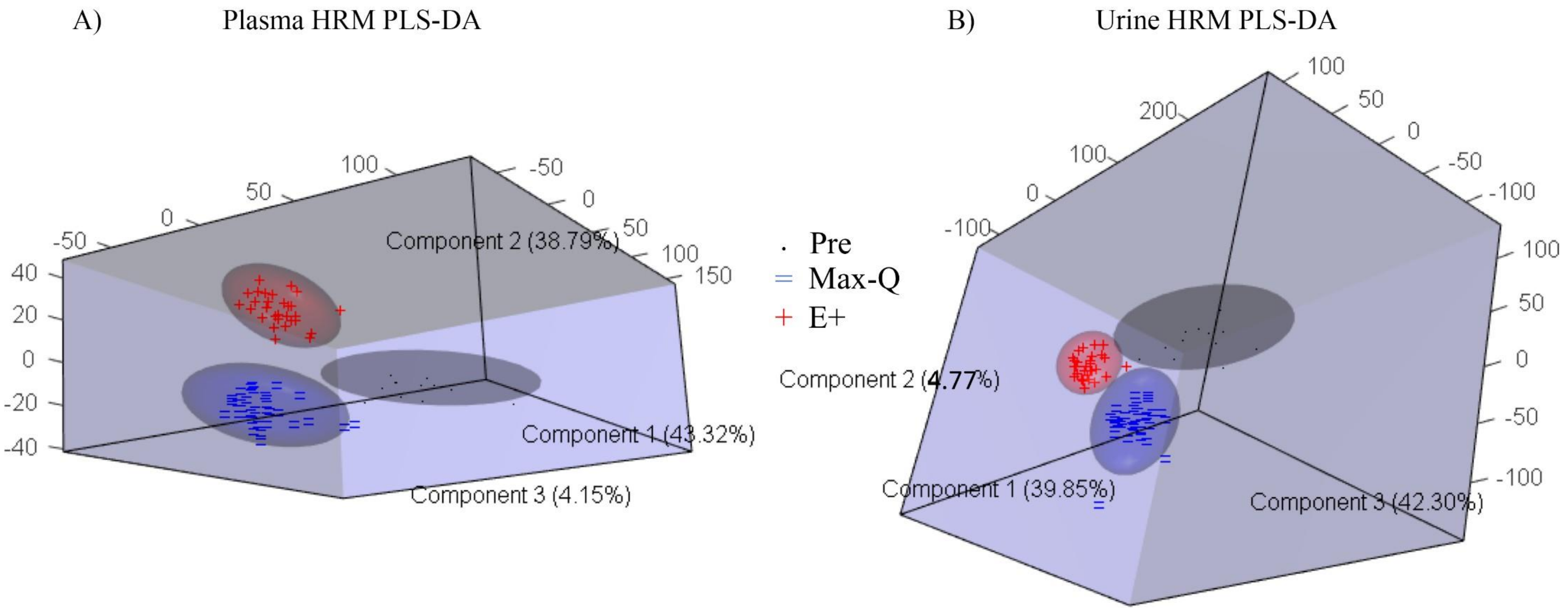

Fig. S3. Partial least squares discriminant analysis (PLS-DA) plots analyzing the fecal microbiota of Angus steers before pasture placement (blue) or grazing either a novel, non-toxic (Max-Q; n = 6; green) or toxic (E+; n = 6; red) endophyte-infected tall fescue over the course of a 26-day grazing trial in the **(A)** plasma and **(B)** urine.

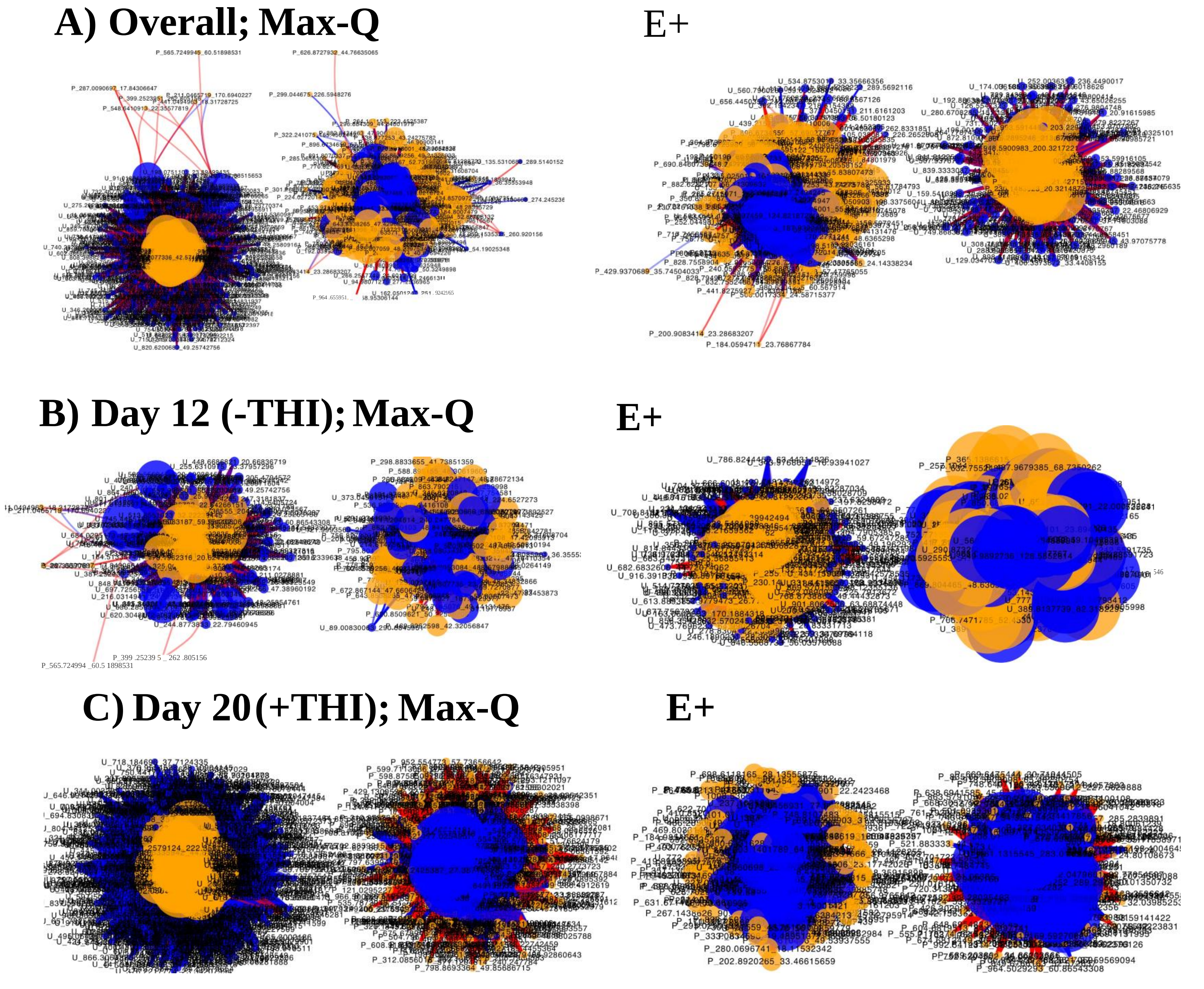
A) Overall; Max-Q
E+
B) Day 12 (-THI); Max-Q
E+
C) Day 20 (+THI); Max-Q
E+

Figure S4. Bipartite network of plasma (orange) and urine (blue) high- resolution metabolomics (HRM) features that were selected by sparse partial least squares regression (sPLS; top 100 plasma and urine HRM features [X and Y matrix, respectfully]) using the *mixOmics* R package and were highly correlated ($|r| > 0.7$) for steers grazing a: **(A)** novel, non-toxic tall fescue (Max-Q) or toxic tall fescue (E+) throughout the grazing trial; **(B)** Max-Q or E+ tall fescue on Day 12 (-THI); **(C)** Max-Q or E+ tall fescue on Day 20 (+THI). Size of the node is respective of the metabolites between centrality within the respective networks.
